# Clinical effects of neoadjuvant chemoradiotherapy versus neoadjuvant chemotherapy on complications and recurrence in patients with advanced gastric cancer

**DOI:** 10.12669/pjms.40.7.9052

**Published:** 2024-08

**Authors:** Rui Wang, Meng Li, Yanjie Zheng, Wenbo Zhang, Ji Song, Fang Yang

**Affiliations:** 1 Rui Wang. Department of Chemoradiotherapy Center, Chengde Central Hospital, Chengde 067000, Hebei, China; 2 Meng Li. Department of Chemoradiotherapy Center, Chengde Central Hospital, Chengde 067000, Hebei, China; 3 Yanjie Zheng. Department of Chemoradiotherapy Center, Chengde Central Hospital, Chengde 067000, Hebei, China; 4 Wenbo Zhang. Department of Chemoradiotherapy Center, Chengde Central Hospital, Chengde 067000, Hebei, China; 5 Ji Song. Department of Chemoradiotherapy Center, Chengde Central Hospital, Chengde 067000, Hebei, China; 6 Fang Yang. Department of Chemoradiotherapy Center, Chengde Central Hospital, Chengde 067000, Hebei, China

**Keywords:** Advanced gastric cancer, Neoadjuvant chemoradiotherapy, Neoadjuvant chemotherapy, Complication

## Abstract

**Objective:**

To compare the clinical effects of neoadjuvant chemoradiotherapy (NCRT) and neoadjuvant chemotherapy (NCT) on complications and recurrence in patients with advanced gastric cancer (AGC).

**Method:**

This was a retrospective study. A total of 83 patients with AGC admitted to Chengde Central Hospital between Jan. 2019 and Jun. 2021 were selected and divided into the observation group(n=41) and the control group(n=42) using a random number table. Patients in the control group received XELOX chemotherapy, and those in the observation group received intensity-modulated radiotherapy (IMRT) with concurrent XELOX chemotherapy. Compared efficacy, pathological complete response rate (pCR), R0 resection rate, adverse reactions, and quality of life (QOL) before and after treatment between the two groups.

**Results:**

The efficacy, pCR, and R0 resection rate of the observation group were significantly increased compared with those of the control group. Comparison of complications showed the number of patients experiencing gastrointestinal (GI) reactions, increased BUN, increased GPT, alopecia, and pigmentation in the observation group was decreased compared with that in the control group, with no statistically significant differences(p>0.05), and the number of patients experiencing myelosuppression was statistically significant between the two groups(p<0.05). There were no significant differences in sub-scores of physical, role, emotional, cognitive, and social functions and the overall score of QOL between the two groups(p>0.05) before treatment.

**Conclusion:**

NCRT is safer and more effective in patients with AGC compared with NCT, and can significantly improve the QOL of patients. It can be widely used in clinical practice.

## INTRODUCTION

Gastric cancer (GC) is a malignant tumor with the highest incidence among malignancies in China.^1^ GC may occur in various parts of the stomach. As a hidden disease, there are no well-defined symptoms in the early stage of GC, and it is mostly diagnosed in the middle and late stages.^2^ AGC is a stage of GC in which the depth of invasion of cancer tissue exceeds the level of submucosa.^3^ In addition, it can be further divided into subtypes of polypoid, local ulcer, infiltrative ulcer, and diffuse infiltration. The treatment of AGC is extremely difficult, and the efficacy of surgical treatment is not satisfactory, with a relatively high postoperative recurrence.^4^ Surgical approaches such as simply expanding the scope of lymph node dissection and combined organ resection cannot provide extra benefits to patients.^5^

Studies have confirmed that previous chemotherapy and radiotherapy can improve the R0 resection rate of patients with local AGC.^6^,^7^ Meanwhile, several studies have demonstrated that previous chemotherapy plays an important role in the treatment of local AGC.^8^-^10^ Many studies on NCT for local AGC have been currently reported, with few studies on NCRT, and even fewer studies on the comparison of the efficacy between the two regimens reported.^11^ Therefore, clinical data of 83 patients with AGC were retrospectively analyzed, compared and analyzed the therapeutic effects of NCRT and NCT on AGC in the present study.

## METHODS

This was a retrospective study. Eighty-three patients with AGC admitted to Chengde Central Hospital between January 2019 and June 2021 were selected by convenience sampling, and divided into the observation group(n=41) and the control group(n=42) based on the treatment regimen they received. The two groups of patients were comparable, with no significant difference in their general information(P>0.05) (Table-I).

**Table-I:** Comparison of general information between the two groups of patients.

	Observation group(n=41)	Control group(n=42)	t/χ^2^	P
Sex			0.006	0.938
Male	27(65.85)	28(66.67)		
Female	14(34.15)	14(33.33)		
Age	56.15±3.71	55.45±4.46	0.776	0.440
***Clinical stages***			0.137	0.934
IIIA	14(34.15)	15(35.71)		
IIIB	21(51.22)	22(52.38)		
IIIC	6(14.63)	5(11.9)		
***Lesion sites***			0.105	0.949
Gastric antrum	22(53.66)	24(57.14)		
Cardia and fundus	17(41.46)	16(38.10)		
Gastric body	2(4.88)	2(4.76)		
***Pathology***			0.122	0.727
Adenocarcinoma	34(82.93)	36(85.71)		
Signet-ring cell carcinoma	7(17.07)	6(14.29)		

### Ethical Approval

The study was approved by the Institutional Ethics Committee of Chengde Central Hospital (No.:2017-15; Date: March 15, 2017), and written informed consent was obtained from all participants.

### Inclusion criteria:

- Patients were diagnosed with AGC by preoperative pathological examination, confirmed by gastroscopy, B-ultrasound, and CT, with no distant metastasis.
- Patients with confirmed local AGC in stages IIIA to IIIC.
- Patients with complete clinical data.
- Patients with no cognitive abnormalities.
- Patients with consciousness.

### Exclusion criteria:

- Concomitant severe infections.
- Other major organ dysfunctions.
- Severe systemic immune system disorders.
- Concomitant mental disorders.
- Contraindications to radiotherapy, chemotherapy, and surgery.

### Methods

Patients in the control group received NCT with XELOX regimen before surgery, including oxaliplatin 130 mg/m^2^ on Day 1 + capecitabine 1 g /m^2^ (twice daily on Day 1-14), every three weeks. Chemotherapy continued for four cycles, and D2 radical surgery was performed 4-6 weeks after chemotherapy.

Patients in the observation group received NCRT. Patients were placed in a supine position, with gastric empty before radiotherapy, and 250 ml of milk was orally administered. Enhanced CT scans were performed with a thickness of 3 mm. The upper boundary of the scan was 3 cm above the diaphragm, and the lower boundary was at the level of the 5th lumbar vertebrae. Intensity modulated radiotherapy (IMRT) was used for radiation therapy using Varian 21EX linear accelerator with 6 MV X-ray, once daily from Monday to Friday, for a total of 5-6 weeks. The chemotherapy regimen was the same as the control group, and D2 radical surgery was performed 6-8 weeks after radiotherapy.

### Outcome Measures:

Treatment evaluation: CR: the disappearance of all lesions at least four weeks; PR: ≥ 30% decrease in the sum of the longest diameter(LD) of the lesions for at least four weeks; SD: the decrease of the sum of LD of the lesions was between PR and PD; PD: ≥20% increased in the sum of LD of the lesions or presence of new lesion(s); and overall response rate (ORR) = (PR + CR)/total patients x100%

Adverse reactions: adverse reactions were classified into 5 grades, including grades 0, 1, 2, 3, and 4; quality of life: the quality of life scale(QOLS) was used to evaluate the physical, emotional, role, cognitive, and social domains of the patients, and a higher score indicated a higher QOL.

### Statistical Analysis

Data were analyzed using SPSS 25.0 software. Measurement data with a normal distribution were presented as *χ̅*±*S*, and t-test was conducted. An independent sample t test was used, categorical variables were presented as n, and %, and χ^2^ test was conducted. Differences with p<0.05 were considered statistically significant.

## RESULTS

The ORR was 92.68% in the observation group, which was significantly increased compared with that in the control group (69.0%), with a statistically significant difference (p<0.05) (Table-II).

**Table-II:** Comparison of efficacy between the two groups [n(%)].

Groups	n	CR	PR	SD	PD	ORR
Observation	41	13(31.71)	25(60.98)	2(4.88)	1(2.44)	38(92.68)
Control	42	3(7.14)	26(61.9)	5(11.9)	8(19.05)	29(69.05)
*χ* ^2^	-	-	-	-	-	7.448
*P*	-	-	-	-	-	0.006

The number of patients experiencing GI reactions, increased BUN, increased GPT, alopecia, and pigmentation in the observation group were decreased compared with that in the control group, and the differences, however, were not statistically significant(p>0.05). The number of patients experiencing myelosuppression in the observation group was reduced compared with that in the control group, with a statistically significant difference(p<0.05) (Table-III).

**Table-III:** Comparison of adverse reactions between the two groups [n(%)].

Adverse reactions	The observation group(n=41)				The control group(n=42)				χ^2^	P

	Grade I	Grade II	Grade III	Grade IV	Grade I	Grade II	Grade III	Grade IV		
GI reactions	5	4	1	0	8	7	3	0	3.165	0.075
Myelosuppression	2	5	1	0	6	10	3	0	6.256	0.012
Increased BUN	7	2	0	0	8	2	1	0	0.204	0.652
Increased GPT	19	9	0	0	20	10	0	0	0.097	0.756
Alopecia	6	10	0	0	7	12	1	0	0.624	0.430
Pigmentation	4	2	0	0	5	1	1	0	0.065	0.799

There were no significant differences in subscores of physical, role, emotional, cognitive, and social functions and the overall score of QOL between the two groups before treatment (p>0.05). The subscores of physical, role, emotional, cognitive, and social functions and the overall score of QOL of the two groups were significantly increased after treatment compared with those before treatment, and these scores after treatment were significantly increased in the observation group compared with those in the control group, with statistically significant differences (p<0.05) (Table-IV). The pCR and R0 resection rates in the observation group were significantly increased compared with those in the control group, with statistical significance (p<0.05) (Table-V).

**Table-IV:** Comparison of QOL before and after treatment between the two groups (*χ̅*±*S*).

Groups	Timepoints	Physical	Role	Emotional	Cognitive	Social	Total
Observation (41)	Before treatment	80.25±4.27	63.53±3.35	63.61±3.38	81.25±3.47	50.53±3.35	50.25±4.47
	After treatment	90.65±4.36	82.38±3.22	81.15±3.26	91.15±4.66	62.78±3.22	68.15±4.66
	t1	10.846	26.102	23.716	10.761	16.868	17.663
	P1	<0.001	<0.001	<0.001	<0.001	<0.001	<0.001
Control (42)	Before treatment	80.21±4.28	63.42±3.38	63.68±3.37	81.81±3.58	50.42±3.38	50.81±4.58
	After treatment	84.52±4.53	71.75±3.41	70.42±3.33	85.45±3.46	56.15±3.71	55.45±4.46
	t1	4.391	10.841	9.056	4.536	7.299	4.630
	P1	<0.001	<0.001	<0.001	<0.001	<0.001	<0.001
Comparison between groups before treatment	t2	0.063	0.053	0.053	0.751	0.226	0.527
	P2	0.950	0.958	0.958	0.455	0.821	0.599
Comparison between groups after treatment	t3	6.299	14.546	14.663	6.253	8.685	12.684
	P3	<0.001	<0.001	<0.001	<0.001	<0.001	<0.001

**Table-V:** Comparison of pCR and R0 resection rates between the two groups [n(%)].

	Observation group (n=41)	Control group (n=42)	χ^2^	P
pCR	13(31.71)	3(7.14)	0.909	0.005
IIIA	7(17.07)	2(4.76)	-	-
IIIB	4(9.76)	1(2.38)	-	-
IIIC	2(4.88)	0(0)	-	-
R0 resection rates	27(65.85)	15(35.71)	7.54	0.006
IIIA	10(24.39)	5(11.9)	-	-
IIIB	13(31.71)	7(16.67)	-	-
IIIC	4(9.76)	3(7.14)	-	-

## DISCUSSION

In the present study, the ORR of the observation group was significantly increased compared with that of the control group (92.68% vs. 69.05%; *p*<0.05); and the pCR and R0 resection rates in the observation group were significantly increased compared with those in the control group (31.71% vs. 7.14%; 65.85% vs. 35.71%, p<0.05). The possible reasons may involve the degraded clinical stage by preoperative radiotherapy. Meanwhile, a comparison of the adverse reactions between the two groups revealed that the adverse reactions of the observation group were less than those of the control group, with no statistically significant differences between the two groups except for myelosuppression. In addition, the QOL of the patients in the observation group was significantly better than that of the control group. This may be due to the fact that the treatment course of chemotherapy of the observation group was shorter and consequently the toxicity was less than that of the control group, although the same chemotherapy regimen was used in both groups and the efficacy, however, was not reduced. Therefore, these results also confirmed that NCRT is safer and more effective with less adverse reactions in patients with AGC compared with NCT, and can significantly improve the QOL of patients with ensured therapeutic effects.

Gastric cancer is commonly encountered in clinical practice, and most patients are diagnosed with AGC, with a high mortality rate, which seriously threatens the physical and mental health of patients and affects their QOL.^12^ Patients with AGC fail to respond to surgery but are relatively sensitive to chemotherapy.^13^ Initial radiotherapy and chemotherapy are important treatments for AGC. NCT refers to systemic chemotherapy before local treatment. Studies have confirmed that NCT can reduce the clinical stage of tumors, improve the overall rate of surgical resection, reduce the intraoperative spread of cancer cells, eliminate potential micrometastases, and block the proliferation of free cancer cells to reduce postoperative metastasis and recurrence.^14^ Moreover, NCT can assist clinicians in understanding the sensitivity of tumors to chemotherapy drugs and choosing sensitive drugs reasonably.

Oxaliplatin is one of the third generation of platinum-based drugs, and its cytotoxicity is stronger than that of cisplatin. Oxaliplatin can bind to the DNA chains and improve performance by blocking the replication and transcription of DNAs in tumor cells. Chemotherapy drugs fluorouracil and 5-fluorouracil(5-FU) have synergistic anti-tumor effects^15^ and are widely used in the treatment of AGC. Capecitabine, a new prodrug form of 5-FU, is converted into inactive 5’-deoxy-5-fluorocytidine after oral administration, which is converted into 5’-deoxy-5-fluorouridine by cytidine deaminase in hepatocytes and tumor cells, and selectively converted into fluorouracil. Capecitabine is rapidly absorbed in the liver with a strong anti-cancer effect on tumor tissues.^16^,^17^ In addition, compared with intravenous administration, oral administration of capecitabine is associated with various advantages such as reduced pain in patients.^18^

Previous chemotherapy can improve the survival of patients with local AGC. However, the survival time prolonged is limited and recurrence or metastasis still occurs in most patients, with a five years overall survival rate (OS) of less than 40%. Therefore, efforts were made to combine radiotherapy on the basis of previous chemotherapy to further improve the survival rate of patients with local AGC. In the study by LIU et al.^19^, 36 patients with AGC or complex esophageal adenocarcinoma received preoperative radiotherapy and two cycles of SOX chemotherapy. The postoperative pCR rate was 13.9%, and the three years follow-up survival was 30.3 months, indicating that preoperative radiotherapy and chemotherapy can improve the three years disease-free survival rate of patients with local AGC or gastric adenocarcinoma. Relevant studies have shown that the simultaneous and continuous use of radiotherapy and chemotherapy may provide survival benefits for some patients with local AGC.^20^

### Limitations

However, this study also has some shortcomings, such as small sample size, small course of treatment, and short follow-up time. It still needs further clinical research to observe the long-term clinical effect of neoadjuvant chemoradiotherapy on progression of gastric cancer, so as to apply a better scheme to patients in need.

## CONCLUSIONS

NCRT is safer and more effective in patients with AGC compared with NCT, and can significantly improve the QOL of patients. It can be widely used in clinical practice.

### Authors’ Contributions:

**RW** and **FY:** Carried out the studies, data collection, drafted the manuscript, are responsible and accountable for the accuracy and r integrity of the work.

**ML** and **YZ:** Performed the statistical analysis and participated in its design.

**WZ** and **JS:** Performed the statistical analysis and participated in its design.

All authors read and approved the final manuscript.
